# Supplementary figures and images for: The Effect of a New N-hetero Cycle Derivative on Behavior and Inflammation against the Background of Ischemic Stroke
Source: Molecules. 2022 Aug 26;27(17):5488. doi: 10.3390/molecules27175488 (PMC9457934; doi:10.3390/molecules27175488)

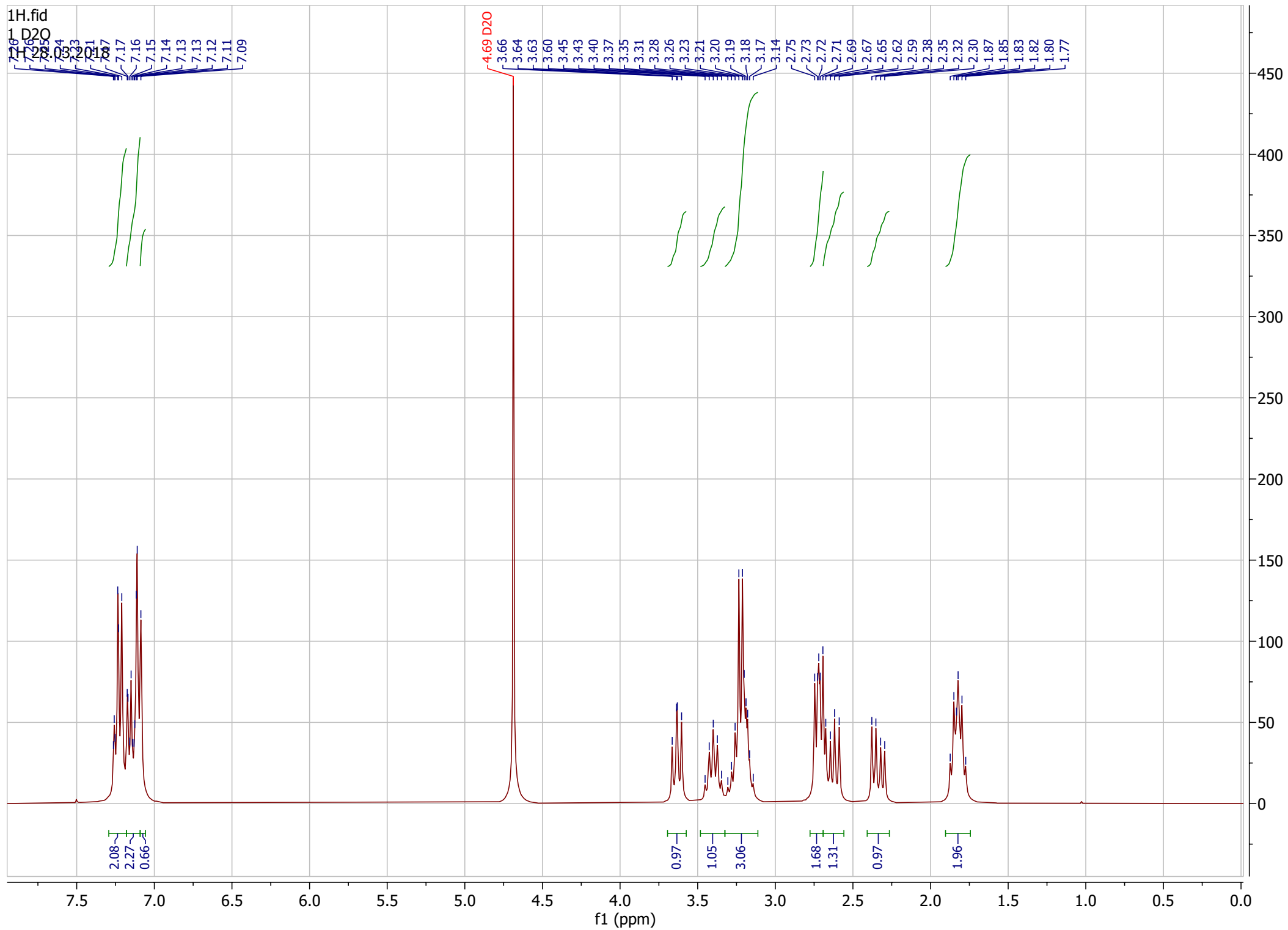

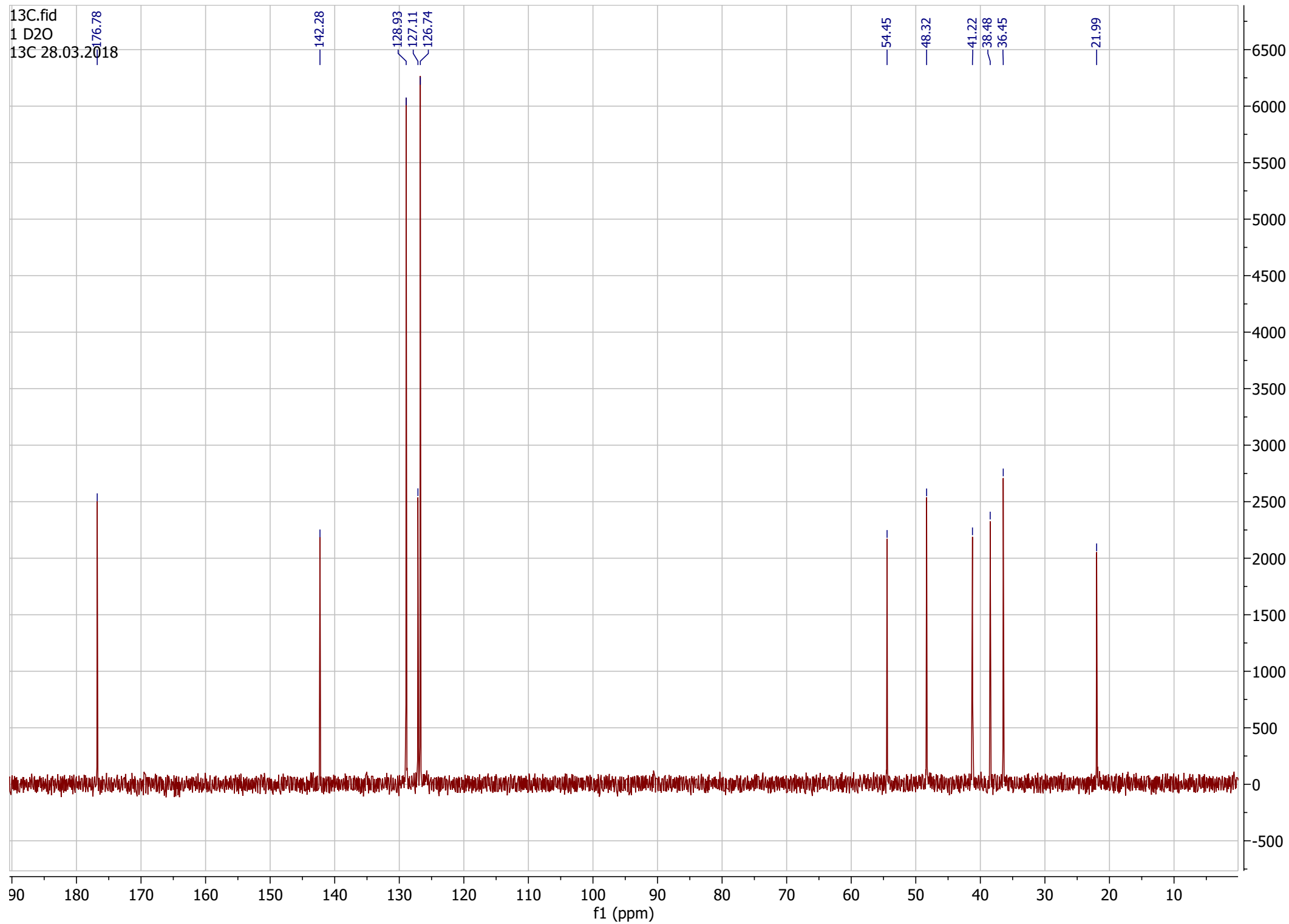

Supplement: Supplementary file 1 [file molecules-27-05488-s001.zip › molecules-1853680-supplementary.pdf]
